# Metabotropic glutamate receptor subtype 2 is a cellular receptor for rabies virus

**DOI:** 10.1371/journal.ppat.1007189

**Published:** 2018-07-20

**Authors:** Jinliang Wang, Zilong Wang, Renqiang Liu, Lei Shuai, Xinxin Wang, Jie Luo, Chong Wang, Weiye Chen, Xijun Wang, Jinying Ge, Xijun He, Zhiyuan Wen, Zhigao Bu

**Affiliations:** 1 State Key Laboratory of Veterinary Biotechnology, Harbin Veterinary Research Institute, Chinese Academy of Agricultural Sciences, Harbin, P. R. China; 2 Jiangsu Co-innovation Center for Prevention and Control of Important Animal Infectious Diseases and Zoonoses, Yangzhou University, Yangzhou, P. R. China; Thomas Jefferson University, UNITED STATES

## Abstract

Rabies virus (RABV) invades the central nervous system and nearly always causes fatal disease in humans. How RABV interacts with host neuron membrane receptors to become internalized and cause rabid symptoms is not yet fully understood. Here, we identified a novel receptor of RABV, which RABV uses to infect neurons. We found that metabotropic glutamate receptor subtype 2 (mGluR2), a member of the G protein-coupled receptor family that is abundant in the central nervous system, directly interacts with RABV glycoprotein to mediate virus entry. RABV infection was drastically decreased after mGluR2 siRNA knock-down in cells. Antibodies to mGluR2 blocked RABV infection in cells *in vitro*. Moreover, mGluR2 ectodomain soluble protein neutralized the infectivity of RABV cell-adapted strains and a street strain in cells (*in vitro)* and in mice (*in vivo)*. We further found that RABV and mGluR2 are internalized into cells and transported to early and late endosomes together. These results suggest that mGluR2 is a functional cellular entry receptor for RABV. Our findings may open a door to explore and understand the neuropathogenesis of rabies.

## Author summary

Rabies virus (RABV) invades the central nervous system and nearly always causes fatal disease in humans. How RABV interacts with host neuron membrane receptors to become internalized and cause rabid symptoms is not yet fully understood. Here, we identified metabotropic glutamate receptor subtype 2 (mGluR2) as a novel cellular RABV receptor for host cell entry. mGluR2 directly interacts with RABV G protein and both proteins are internalized together. Our findings may open a door to explore the fundamental molecular mechanisms for rabies neuropathogenesis.

## Introduction

Rabies is an almost always fatal disease caused by rabies virus (RABV); it still kills about 60,000 people worldwide per year [1], most of whom are children in developing countries. Rabies has the highest case fatality rate among all infectious diseases, imposing a burden that is at the very least comparable to that imposed by other major zoonoses [2]. RABV has a very broad host spectrum; almost all warm-blooded animals can be infected. Humans are usually infected when bitten by RABV-infected dogs, and the virus then invades the central nervous system (CNS) and causes rabies symptoms [3]. About two-thirds of patients suffer from the furious form; the other third experience paralytic symptoms [4]. Once symptoms appear, no treatment is proven to prevent death and the mortality rate is almost 100%. The mystery of RABV neuropathogenesis is still being unraveled despite many years of research, and this lack of information has hindered the development of therapy for rabies.

RABV is a non-segmented negative-stranded RNA virus that belongs to the *Genus Lyssavirus* of the *Family Rhabdoviridae*. The RABV genome consists of five genes that respectively encode five proteins: nucleoprotein (N), phosphoprotein (P), matrix protein (M), glycoprotein (G), and large polymerase protein (L). The G protein is a transmembrane glycoprotein that is responsible for receptor binding during infection [5, 6]. Previous studies suggest that RABV may use different receptors during the various stages of its journey to invade the CNS [7]. To date, three proteins, nAchR (acetylcholine receptor subunit alpha, CHRNA1) [8], NCAM (neural cell adhesion molecule) [9], and p75NTR (low-affinity nerve-growth factor receptor) [10] are proposed to play a role as receptors for RABV entry. nAchR was the first of these proteins to be identified as a RABV receptor. It mainly resides in neuromuscular junctions, and is thought to mediate street virus infection of and replication in muscle cells [7, 11, 12]. NCAM has been shown to be a receptor for a RABV laboratory strain [9], and p75NTR can facilitate RABV infection [10] and retrograde axonal transport [13]. Carbohydrates, gangliosides, and lipids have also been implicated in RABV entry [14–16], but their functions are not well established. After binding, RABV penetration depends on clathrin-mediated endocytosis and acidification in late endosomes [6, 17, 18]. How RABV interacts with host neuron membrane receptors to become internalized and cause symptoms is not yet fully understood.

**Fig 1:**
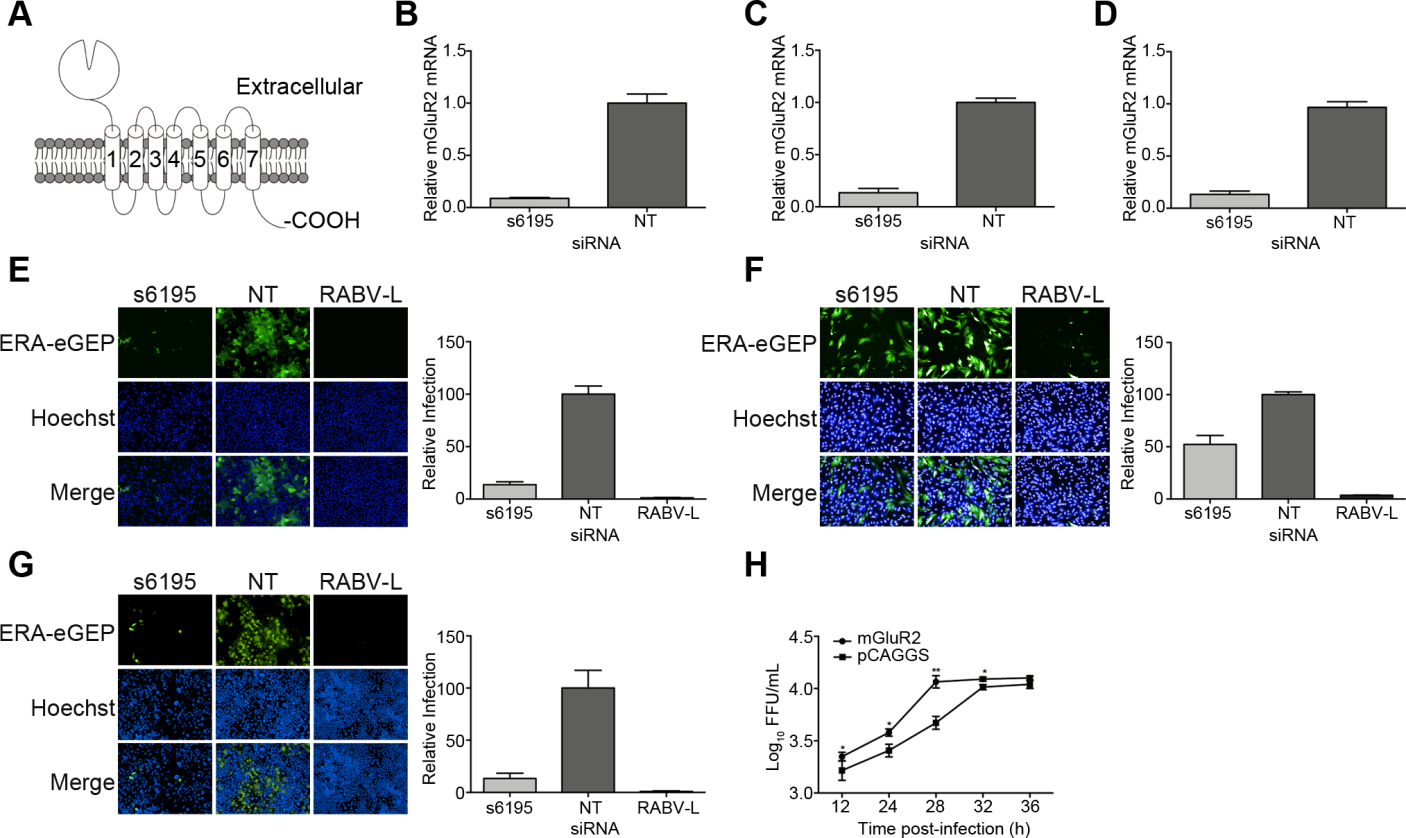
siRNA silencing and overexpression of mGluR2 affect RABV infection of cells. (A) Schematic for mGluR2 protein. Transfection with siRNA s6195 resulted in the downregulation of mGluR2 mRNA in transfected HEK293 cells (B), SK cells (C), and N2a cells (D). Silencing of mGluR2 inhibited ERA-eGFP infection of HEK293 cells (E), SK cells (F), and N2a cells (G). NT, non-targeting siRNA. (H) Overexpression of mGluR2 enhanced the growth of ERA-eGFP at a multiplicity of infection (MOI) of five in transfected HEK293 cells; virus titers in the cell culture supernatant were determined as focus forming units (FFU) in BSR-T7/5 cells. A one-way ANOVA was used for the statistical analysis. *, *p*<0.05. **, *p*<0.01.

Here, we identified metabotropic glutamate receptor 2 (mGluR2), a member of the class C family of G protein-coupled receptors (GPCRs) (Fig 1A), as a cellular receptor for RABV. We found that RABV could specifically bind to mGluR2. Antibodies against mGluR2 blocked RABV infection of cells. The mGluR2 ectodomain soluble protein neutralized RABV infection of cells and mice. Moreover, we found that RABV and mGluR2 are internalized into cells and transported to early and late endosomes together. Collectively, our findings suggest that mGluR2 is a novel cellular receptor for RABV.

## Results

### mGluR2 is essential for RABV infection

We used a global RNAi strategy to screen potential host factors for RABV infection in the human embryonic kidney cell line HEK293 with 64,755 siRNAs targeting 21,585 mRNAs in the human genome (three independent siRNAs for each mRNA). We found that downregulating mGluR2 mRNA significantly inhibited the replication of ERA-eGFP, a recombinant RABV ERA strain that expresses enhanced green florescence protein (ERA-eGFP). We tested mGluR2 expression in HEK293 cells, the human neuroblastoma cell line SK-N-SH (SK), and the mouse neuroblastoma cell line N2a. Cell membrane protein was isolated and subjected to western blotting to detect mGluR2. In the interim, cells were stained for surface mGluR2 expression by flow cytometry. The results demonstrated all three cell types express mGluR2 on the cell surface (S1 Fig). To confirm that mGluR2 is necessary for RABV infection of cells, we knocked-down mGluR2 expression by transfecting HEK293 cells, SK cells, and N2a cells with the siRNA s6195, which targets mGluR2 mRNA. Quantitative RT-PCR analysis showed that mGluR2 mRNA expression was reduced by 90% in all three cell types at 18 h after transfection (Fig 1B, 1C and 1D). Compared to mock-transfected cells, RABV replication titers decreased by 85%, 48%, and 82% in mGluR2-silenced HEK293, SK, and N2a cells, respectively (Fig 1E, 1F and 1G). We also found that mGluR2 overexpression facilitated RABV infection. RABV growth titers were significantly higher in HEK293 cells transfected with mGluR2 cDNA than in mock-transfected cells at various time points post-infection (Fig 1H). These results indicate that mGluR2 is an important host cell factor for RABV infection.

### mGluR2 binds RABV G through a direct interaction

**Fig 2:**
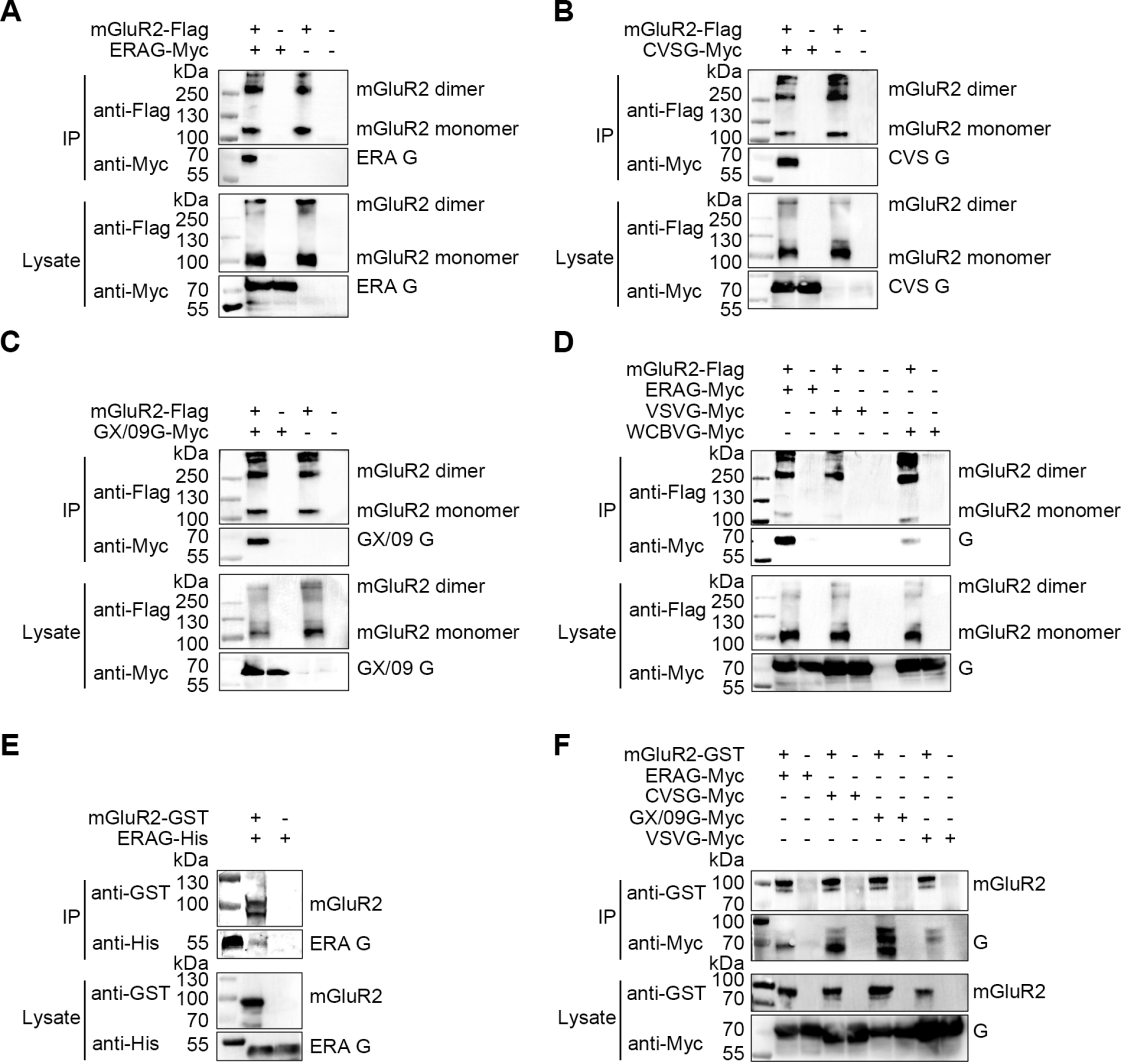
Interactions between RABV G and mGluR2. The mGluR2-Flag interacted with ERAG-Myc (A), CVSG-Myc (B), GX/09G-Myc (C), and WCBVG-Myc but not VSVG-Myc (D) in co-IP assays with plasmid-transfected HEK293 cell lysates. Purified mGluR2-GST pulled down purified ERAG-His (E). Purified mGluR2-GST pulled down ERAG-Myc, CVSG-Myc, and GX/09G-Myc but not VSVG-Myc from lysates of transfected HEK293 cells (F).

mGluR2 is a seven-transmembrane receptor (Fig 1A) and is genetically conserved among different mammalian species. It is abundant in the CNS and very rarely expressed in other tissues [19]. As RABV is the prototypical neurotropic virus, we hypothesized that mGluR2 may serve as a novel receptor for RABV to enter CNS cells. A direct interaction between mGluR2 and RABV G would strongly support the idea that mGluR2 functions as a host receptor for RABV infection. To investigate whether mGluR2 interacts with RABV G, we carried out co-immunoprecipitation (co-IP) and pull-down assays with mGluR2 and RABV G derived from the cell-adapted strain ERA, the mouse-adapted strain CVS-24, street virus GX/09, West Caucasian bat virus (WCBV), another member of the lyssavirus family, and the glycoprotein (G) of vesicular stomatitis virus (VSV), another member of the Rhabdoviridae family. Flag-tagged mGluR2 protein (mGluR2-flag) was co-expressed with Myc-tagged RABV G from the ERA strain (ERAG-Myc), the CVS strain (CVSG-Myc), the GX/09 street virus (GX/09G-Myc), WCBV G (WCBVG-Myc), and VSV G (VSVG-Myc) in plasmid-transfected HEK293 cells for the co-IP assay. All four Lyssavirus G proteins interacted with the mGluR2 protein in its dimeric (~250 kDa) and monomeric (~110 kDa) forms. In contrast, mGluR2 did not interact with VSVG-Myc (Fig 2A–2D). To better control potential nonspecific effects, we used goat SLAM-Flag (signaling lymphocytic activation molecule, serves as a cellular receptor for Peste des petits ruminants virus) and human CD20-Flag (a B-cell cell surface marker) to co-immunoprecipitate with RABVG-Myc. As shown in S2 Fig, RABV G did not interact with SLAM or CD20, further demonstrating the specificity of the interaction between mGluR2 and RABV G.

To determine whether mGluR2 interacts with RABV G directly, we pooled the purified recombinant GST-tagged mGluR2 protein ectodomain (mGluR2-GST) with the purified recombinant His-tagged RABV G ectodomain derived from the ERA strain (ERAG-His) for a pull-down assay. We found that ERAG-His was successfully pulled-down by mGluR2-GST (Fig 2E), thereby confirming that ERA G interacted with mGluR2 directly. We also demonstrated that purified mGluR2-GST could pull-down recombinant CVSG-Myc, GX/09G-Myc, and WCBVG-Myc in plasmid-transfected HEK293 cells, but failed to pull-down VSVG-Myc (Fig 2F). These results confirm that mGluR2 interacts directly with RABV G.

### mGluR2 antibodies block RABV infection in cells

**Fig 3:**
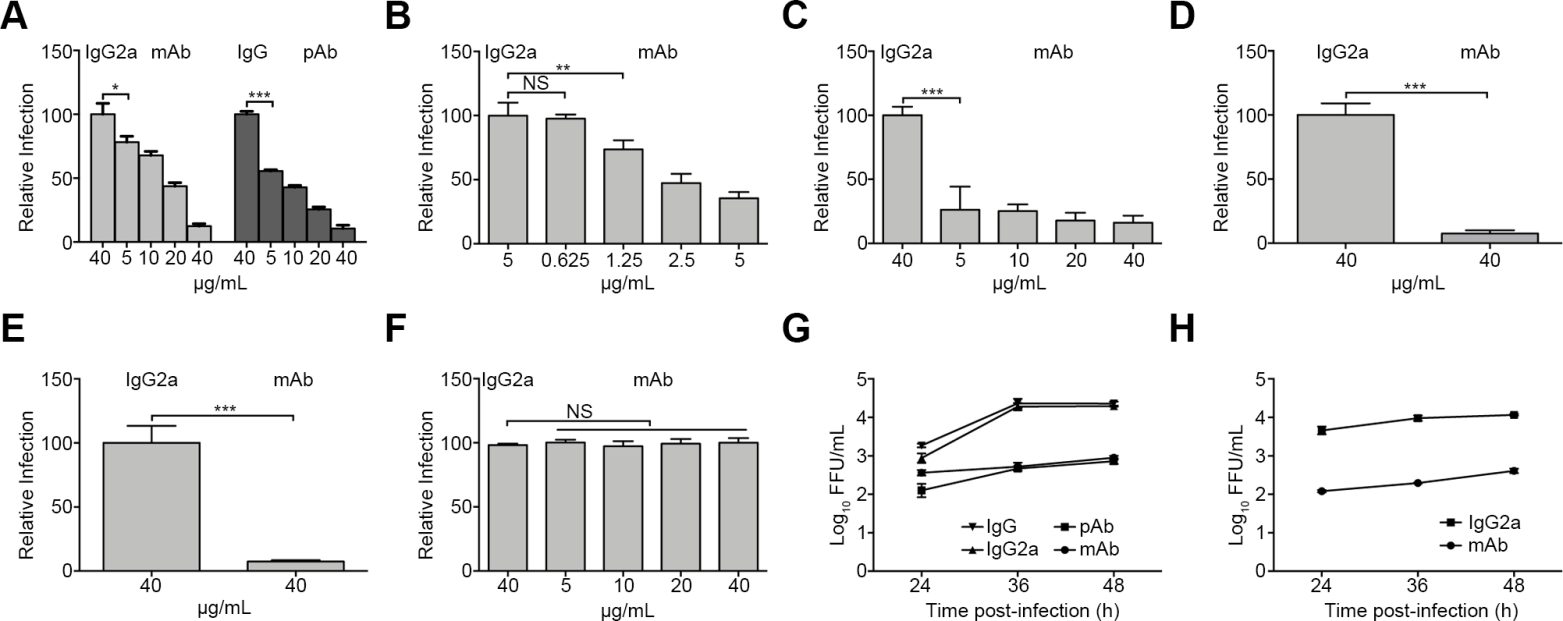
Antibodies to mGluR2 block RABV infection of cells in a dose-dependent manner. The monoclonal antibody (mAb) or polyclonal antibody (pAb) against mGluR2 blocked ERA-eGFP infection of HEK293 cells (A), SK cells (B), N2a cells (C), and mPN cells (D). The mAb against mGluR2 also blocked VSV∆G-ERAG-eGFP infection (E) but failed to block VSV-eGFP infection (F) of HEK293 cells. The mAb or pAb against mGluR2 decreased the replication of ERA-eGFP in HEK293 cells (G) and mPN cells (H); virus titers in the cell culture supernatant were determined as FFU in BSR/T7-5 cells. The isotypes IgG2a and IgG at the highest concentration were used as controls for the mAb and pAb, respectively. A one-way ANOVA was used for the statistical analysis. *, *p*<0.05. **, *p*<0.01. ***, *p*<0.001.

To investigate whether mGluR2 serves as a receptor for RABV infection, we tested whether antibody against mGluR2 could block RABV infection *in vitro*. But first, we tested the cytotoxicity of the mGluR2 antibody in HEK293, SK, N2a and mouse primary neuronal (mPN) cells. The results confirmed that cell viability was unaffected by the mGluR2 monoclonal antibody (mAb) at the highest concentration used in the following infection inhibition assay (S3 Fig). In HEK293 cells, mAb and polyclonal antibody (pAb) inhibited infection by ERA-eGFP. The inhibitory effect started at 5 μg/mL and increased with increasing amounts of antibody (Fig 3A). The mAb against mGluR2 showed a similar inhibitory effect on ERA-eGFP infection of human neural SK cells, mouse neural N2a cells, and mPN cells (Fig 3B, 3C and 3D). Due to the cytotoxicity of the mGluR2 antibody to SK cells, we began with a reduced amount of mAb (5 μg/mL); the results still showed a dose-dependent inhibitory effect on ERA-eGFP infection. The mAb against mGluR2 also inhibited VSV∆G-eGFP-ERAG (Fig 3E), a chimeric VSV in which the open reading frame of the G gene was replaced with that of the G gene of the RABV ERA strain, which was generated as described previously [20]. In contrast, the mAb against mGluR2 had no significant inhibitory effect on infection by VSV-eGFP, a recombinant VSV expressing eGFP (Fig 3F). The isotype antibodies IgG2a and IgG at 40 μg/mL displayed no inhibitory effect on the infections caused by ERA-eGFP, VSV∆G-eGFP-ERAG, or VSV-eGFP in different cell types (Fig 3A–3F). Growth kinetics of ERA-eGFP in the anti-mGluR2 antibody-treated HEK293 and mPN cells were also determined. At different times post-infection, the viral titers from the mAb- or pAb-treated HEK293 cells and mPN cells were significantly lower than those of the corresponding IgG isotype-treated controls (Fig 3G and 3H). These results demonstrate that blocking the cell surface mGluR2 inhibits RABV infection but not VSV infection.

### The mGluR2 ectodomain soluble protein neutralizes RABV infection of cells and mice

**Fig 4:**
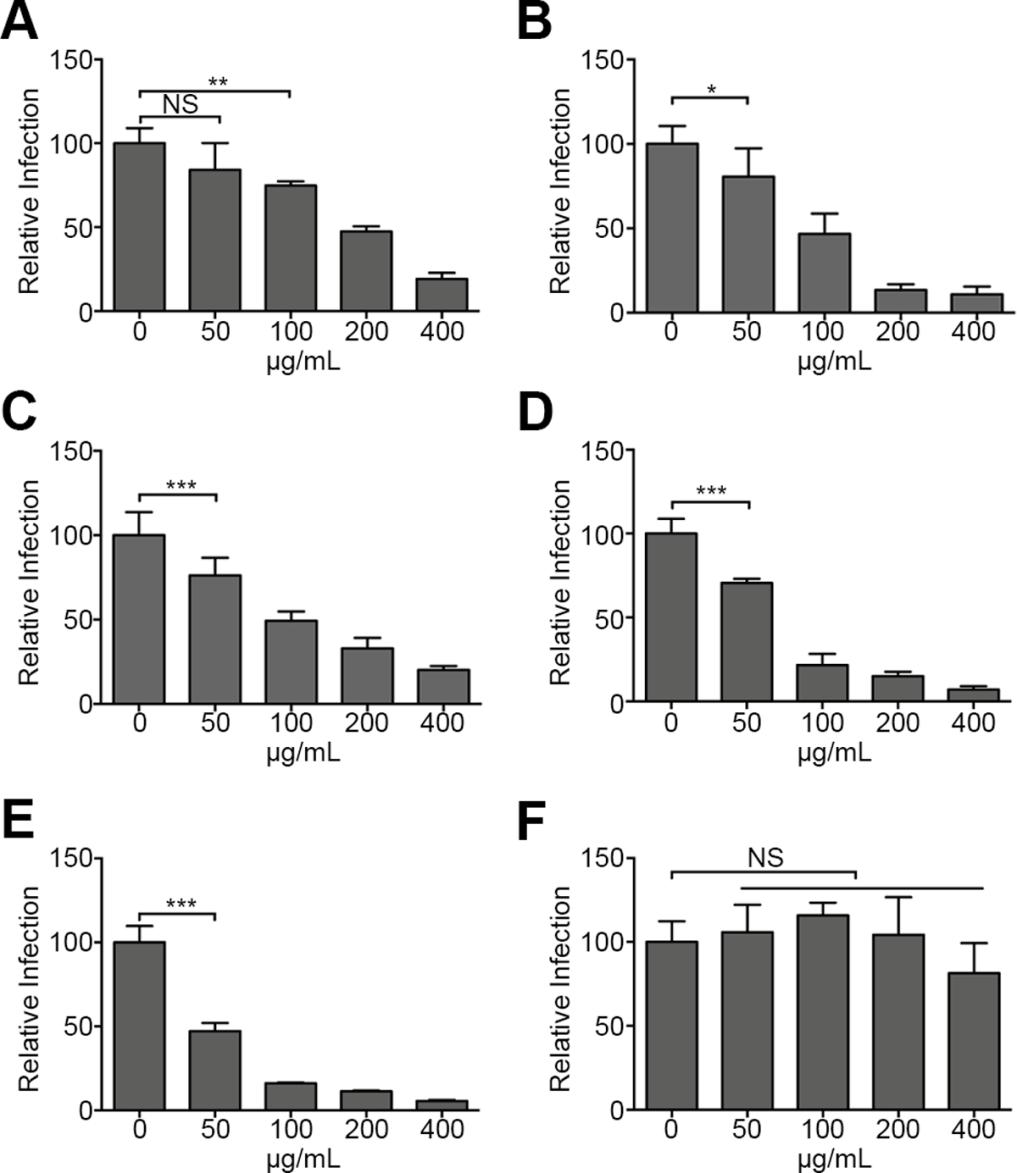
The mGluR2 ectodomain soluble protein (mGluR2-GST) neutralizes the infectivity of RABV in a dose-dependent manner. mGluR2-GST neutralized ERA-eGFP infection of HEK293 cells (A), SK cells (B), N2a cells (C), and mPN cells (D), and neutralized VSV∆G-ERAG-eGFP infection of HEK293 cells (E) but failed to neutralize VSV-eGFP infection of HEK293 cells (F). A one-way ANOVA was used for the statistical analysis. *, *p*<0.05. **, *p*<0.01. ***, *p*<0.001.

If RABV uses mGluR2 as a host cell receptor for infection, the soluble mGluR2 protein should neutralize RABV infection. To test this hypothesis, we performed neutralization assays *in vitro* by using ERA-eGFP and mGluR2-GST. We found that mGluR2-GST neutralized the infectivity of ERA-eGFP in HEK293 cells, SK cells, N2a cells, and mPN cells in a dose-dependent manner (Fig 4A–4D). In HEK293 cells, the 50% inhibitory dose of mGluR2-GST was about 200 μg/mL at 48 h post-infection, whereas for VSV∆G-eGFP-ERAG, it was about 50 μg/mL (Fig 4E). The inhibitory effectiveness of mGluR2-GST in SK cells, N2a cells, and mPN cells was also dose-dependent, with 50% inhibitory doses of about 50 μg/mL, 50 μg/mL, and 50–100 μg/mL, respectively. In contrast, mGluR2-GST had no significant neutralizing effect on VSV-eGFP *in vitro* (Fig 4F).

**Fig 5:**
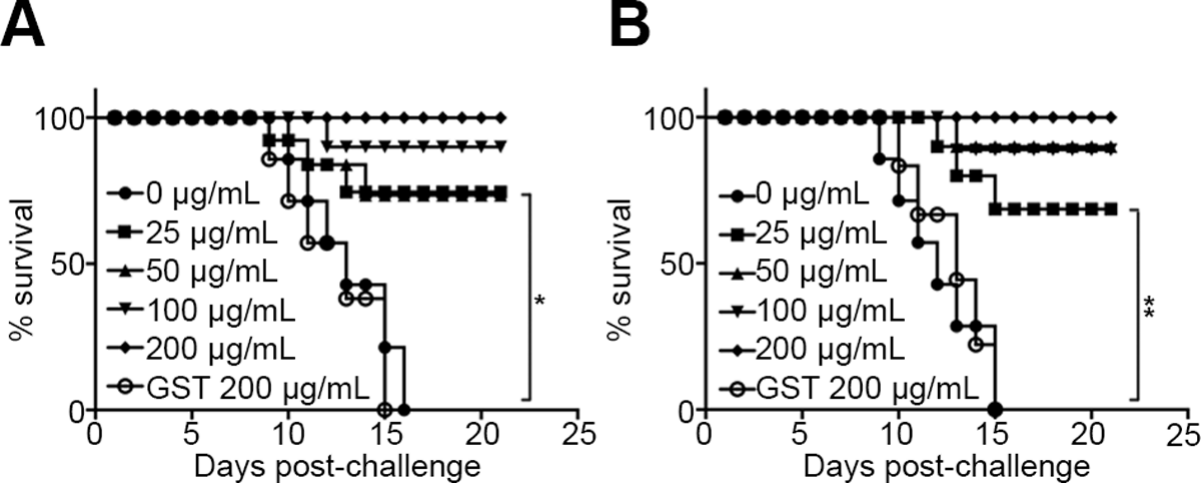
The mGluR2 ectodomain soluble protein (mGluR2-GST) protects mice from lethal challenges in a dose-dependent manner. mGluR2-GST neutralized the infectivity of GX/09 street virus and protected mice from lethal virus challenges *via* intramuscular (A) or intracerebral (B) inoculation. The Log-rank (Mantel-Cox) test was used to analyze the statistical difference between the survival rates of the challenged mice. *, *p*<0.05. **, *p*<0.01.

Because RABV street virus does not replicate efficiently in cells *in vitro*, we performed the neutralization test with the RABV street virus GX/09 in mice. We mixed different amounts of mGluR2-GST (from 25 μg to 200 μg) with a fixed 50% mouse lethal dose (MLD_50_) of RABV GX/09, and then inoculated mice with the mixtures via the intramuscular (*i*.*m*) or intracerebral (*i*.*c*) route. The GX/09 virus doses for challenge via *i*.*m* and *i*.*c* inoculation were 10 MLD_50_ and 5 MLD_50_, respectively. Mice were observed for 21 days for signs of sickness or death. We found that mGluR2-GST neutralized RABV GX/09 and protected mice from lethal challenge in a dose-dependent manner. GST alone showed no protective effect for *i*.*m* and *i*.*c* challenged mice. At a concentration of 200 μg/mL, mGluR2-GST neutralized the infectivity of RABV GX/09, and conferred complete protection to the treated mice, which showed no signs or symptoms of infection following either *i*.*m* or *i*.*c* challenge (Fig 5A and 5B). These results suggest that mGluR2 is a functional receptor for RABV to enter cells.

To further address the importance of mGluR2 *in vivo*, we performed immunohistochemistry (IHC) and immunohistofluorescence (IHF) (S6 Fig) for mGluR2 and RABV antigen in most areas of the brain of mice infected with the street virus GX/09 strain. mGluR2 was distributed throughout the mouse brain (S6A and S6C Fig), although both IHC and IHF displayed uneven staining density or fluorescence intensity across whole brain sections. It was clear that mGluR2 was expressed in different neuron cells with a large variation in levels of expression. There were more RABV antigen-positive cells in the brainstem than in the cerebrum and cerebellum (S6B and S6D Fig). In the cerebrum, the infected cells in medulla were much more than that in cortex. In cerebellum, most of the Purkinje cells were RABV-positive. There were also very few RABV-infected cells in the olfactory bulb. We observed that the distribution pattern of mGluR2 did not exactly match that of RABV antigen. Therefore, we randomly selected five fields from the brainstem (S6I and S6III Fig), the cerebellum (S6II Fig), the cerebral medulla (S6IV Fig) and the olfactory bulb (S6V Fig) for detailed observation. We found that mGluR2 and RABV antigen colocalized in portion of the RABV-infected neuron cells (S6 Fig). Some RABV-infected cells showed mGluR2 IHF-positive staining, whereas some were negative (S6I, S6II, S6III, S6IV and S6V Fig). These results confirmed that mouse brain cells expressing mGluR2 could be infected by street RABV, which further suggests that mGluR2 serves as a receptor for RABV infection *in vivo*.

### RABV and mGluR2 are internalized into cells and transported to early and late endosomes together

**Fig 6:**
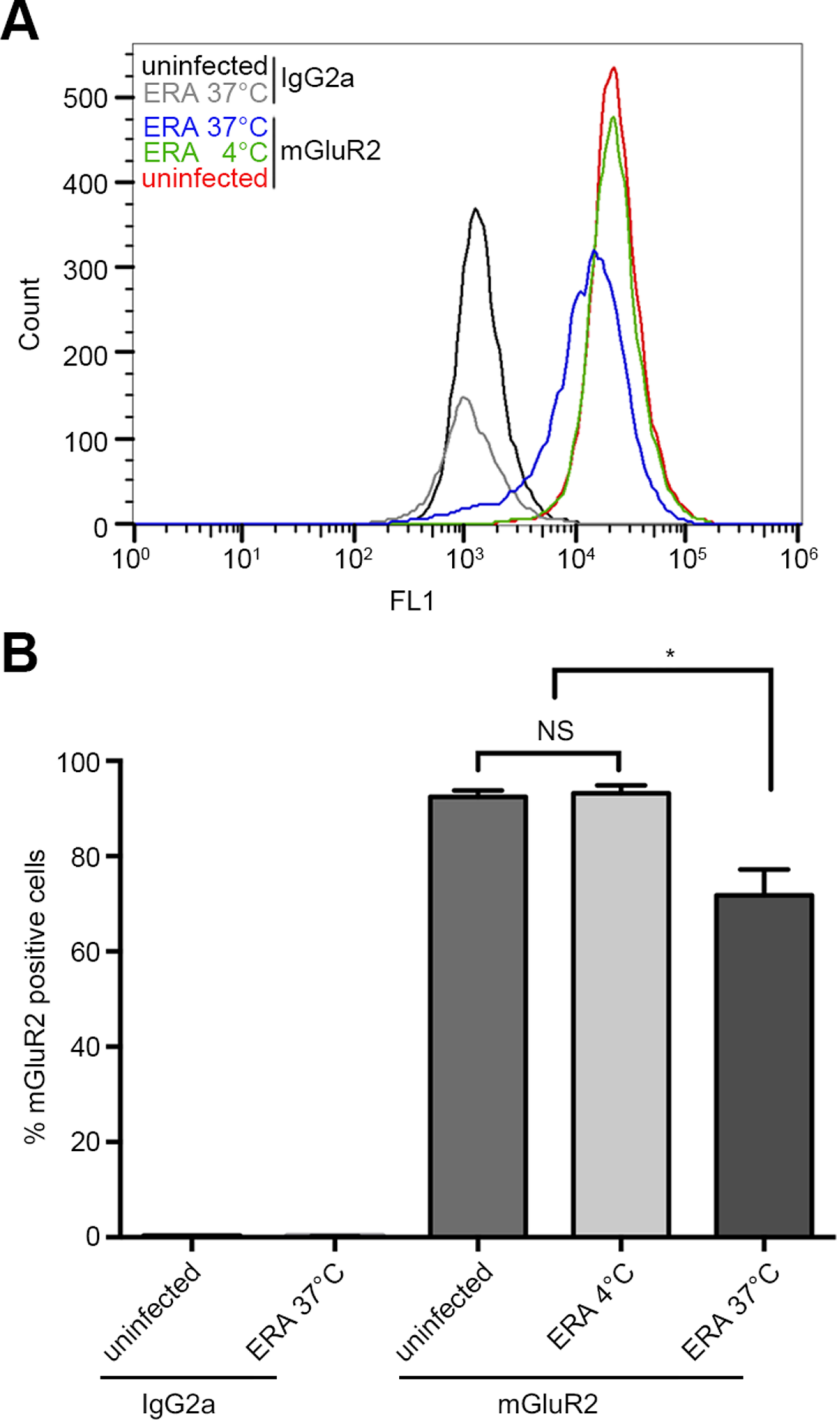
RABV binding results in the downregulation of cell surface mGluR2. Flow cytometry detected cell surface mGluR2 on RABV ERA-infected HEK293 cells (ERA), which was compared to uninfected HEK293 cells by staining with anti-mGluR2 monoclonal antibody (mGluR2) or isotype antibody control (IgG2a) at 4°C or 37°C (A). The flow cytometry results were analyzed with a one-way ANOVA (B). *, *p*<0.05. **, *p*<0.01.

**Fig 7:**
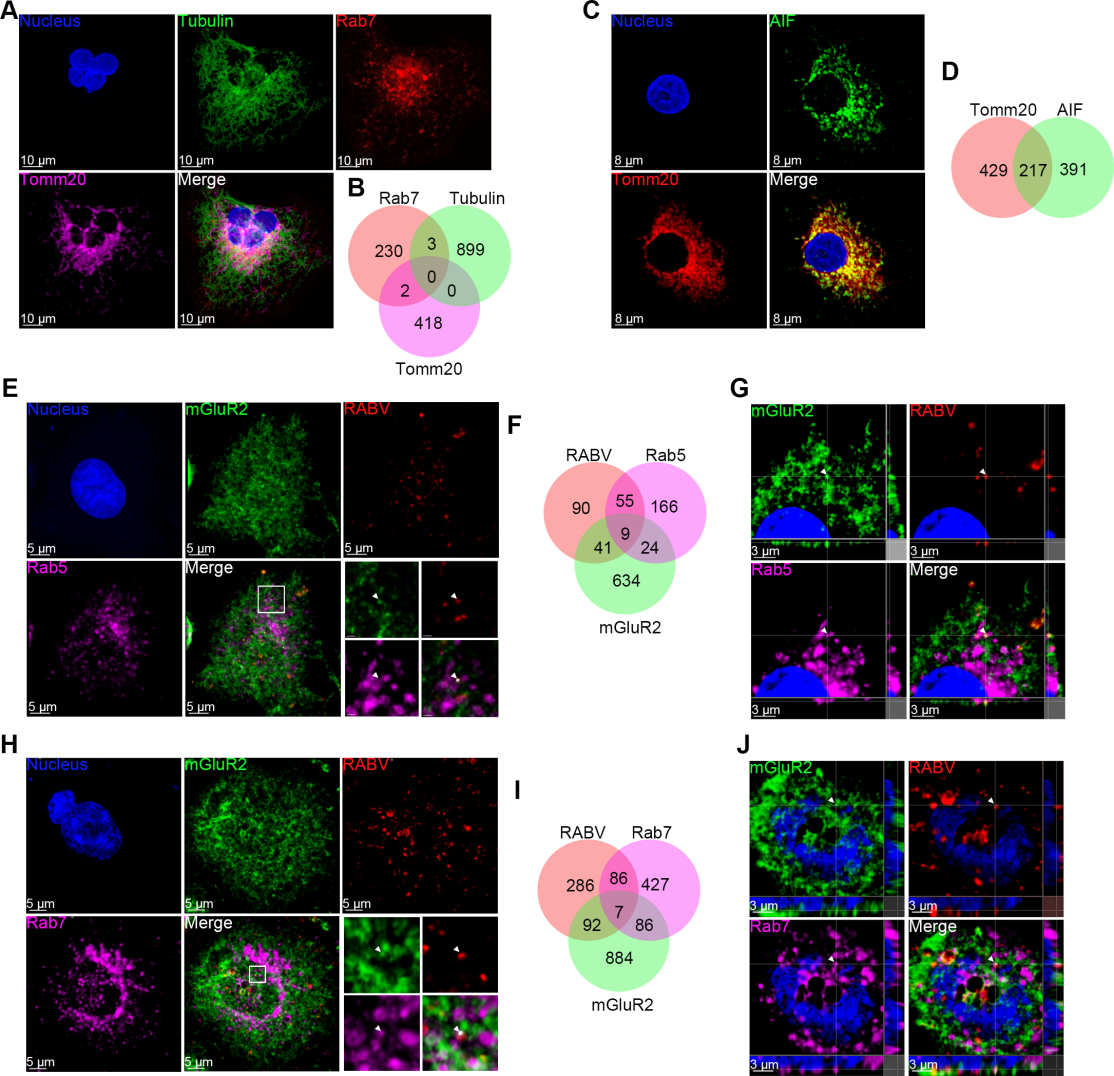
RABV and mGluR2 are internalized into cells and transported together in early and late endosomes. N2a Cells were stained by using the Tyramide Signal Amplification immunofluorescent method. Absence of co-localization of tubulin (green), Rab7 (red), and Tomm20 (purple) served as a negative control for co-localization (A and B). Significant co-localization of the mitochondrial marker AIF (green) and Tomm20 (red) served as a positive control for co-localization (C and D). N2a cells infected with ERA-N-mCherry for 20 minutes at 37°C were used to perform immunofluorescence staining for RABV antigen (red), mGluR2 (green), Rab5 or Rab7 (purple), and the cell nuclei (blue). Co-localization of the RABV-mGluR2 complex with Rab5 (E, F) or Rab7 (H, I) was observed and counted. The images, comprising three single fluorescence channels (G, J), represent amplified random co-localization spots in the merged image within the small white box (G from E, F from J). The 3D-rendered images were generated by using Imaris software (G, J) and the co-localization of the RABV-mGluR2 complex with Rab5 (G) or Rab7 (J) from the three single fluorescence channels is indicated with the white arrowhead.

It has been suggested that RABV enters host cells through clathrin-dependent endocytosis [6, 17, 18]. Given that RABV G directly binds to mGluR2, we next determined whether mGluR2 is internalized with RABV. We first tested the surface expression level of mGluR2 after RABV infection. Flow cytometry results showed that RABV infection led to a significant decrease in the cell surface expression of mGluR2 (Fig 6), indicating that mGluR2 was internalized upon infection. Next, we used multiplex immunofluorescence staining of RABV particles, mGluR2, Rab5, and Rab7 in RABV ERA-N-mCherry-infected N2a cells, and observed the subcellular localization of the internalized RABV-mGluR2 complex. We first set co-localization negative and positive controls to validate the method. For the negative controls, tubulin, Rab7 (a late endosome marker), and mitochondrial import receptor subunit TOM20 homolog (Tomm20) were stained and their co-localization was analyzed and counted. The results showed no co-localization of the three proteins (Fig 7A and 7B). For the positive controls, mitochondria marker allograft inflammatory factor (AIF) and Tomm20 were stained and their co-localization was analyzed and counted. These results showed that more than half of the AIF and Tomm20 co-localized (Fig 7C and 7D). Next, we analyzed and counted the co-localization of RABV, mGluR2, and Rab5 (or Rab7) in N2a cells. The results showed the co-localization (white spots in merged image) of RABV, mGluR2, and Rab5 (Fig 7E and 7F) or Rab7 (Fig 7H and 7I). We then generated a 3D-rendered image by using Imaris software of randomly chosen co-localized spots, which showed that the RABV-mGluR2 complex was co-localized with Rab5 in early endosomes (Fig 7G) and with Rab7 in late endosomes from three dimensions (Fig 7J) from three single fluorescence channels.

**Fig 8:**
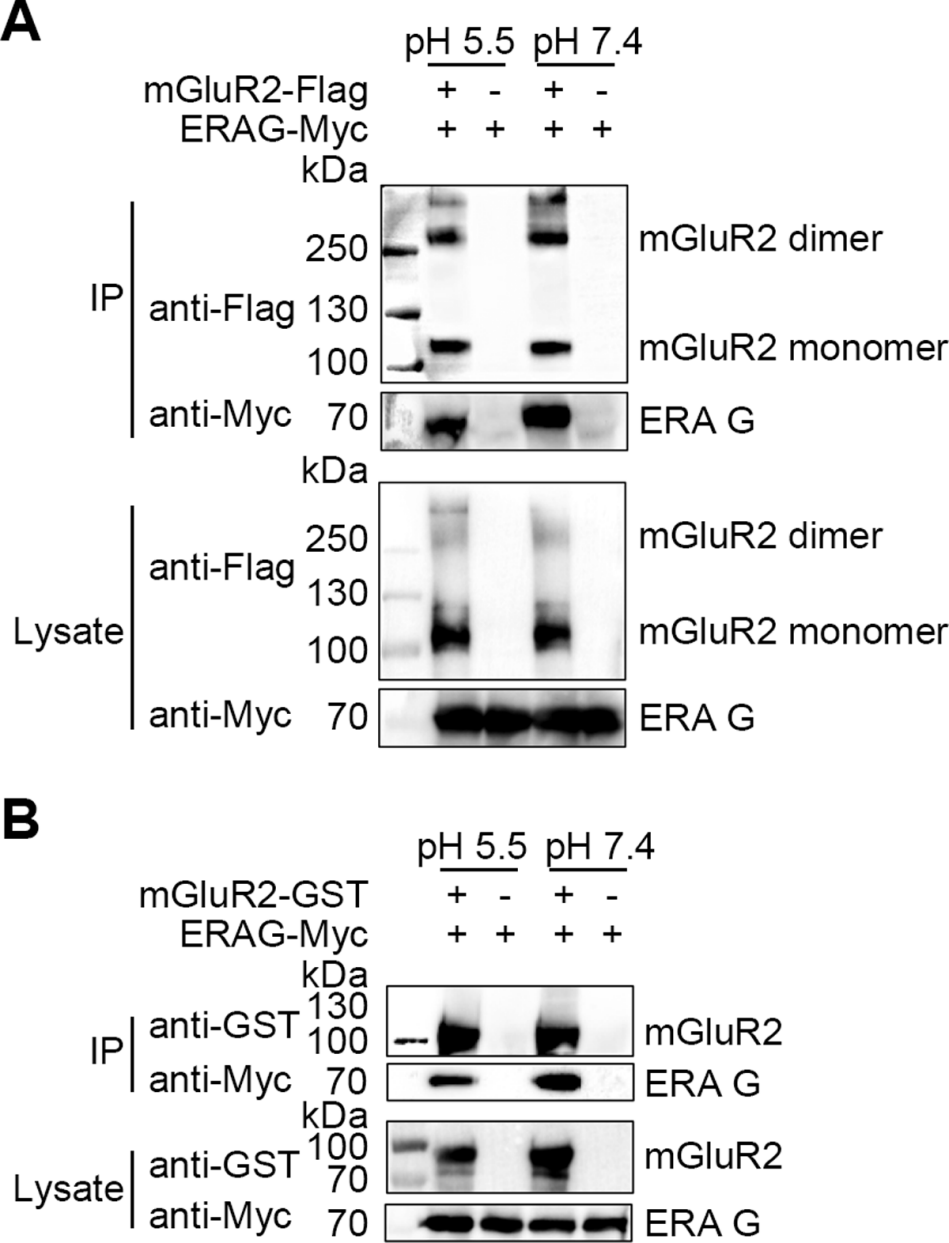
The interaction between RABV G and mGluR2 is retained under lower pH conditions. mGluR2-Flag interacted with ERAG-Myc in co-IP assays with plasmid-transfected HEK293 cell lysates at pH 5.5 and pH 7.2 (A). Purified mGluR2-GST pulled-down ERAG-Myc from the lysates of transfected HEK293 cells at pH 5.5 and pH 7.2 (B).

We also co-immunoprecipitated RABV G and mGluR2 under acidic conditions and showed that the interaction of RABV G with mGluR2 was unaffected by pH 5.5 (Fig 8), which is consistent with our observation that the RABV-mGluR2 complex is present in late endosomes. These results demonstrate that RABV and mGluR2 are internalized into cells and transported together within the cellular endosomal compartments.

## Discussion

Using a global RNAi strategy in the human cell line HEK293, we found that mGluR2 is an important host cell factor for RABV infection. The results *in vitro* from siRNA silencing, protein interaction, antibody blocking, soluble protein neutralization and 3D-rendered image, and *in vivo* from soluble protein neutralization and immunohistofluorescence assays strongly suggest that mGluR2 is a novel cellular receptor for RABV infection. mGluR2 is recognized directly by RABV G but not VSV G, indicating that RABV may be unique among members of the *Rhabdoviridae* family in using this host cell receptor and internalization mechanism.

RABV has evolved to minimize damage to its surroundings while spreading efficiently through the CNS, and selectively causes furious or paralytic symptoms in rabies patients [4, 21], although the mechanisms involved remain largely unknown. mGluR2 is a seven-transmembrane receptor (Fig 1A) that is genetically conserved among different mammal species. It is abundant in the CNS and very rarely expressed in other tissues [19]. mGluR2 has been linked to the inhibition of the cAMP cascade and is activated by L-glutamate to mediate fast synaptic responses [22, 23]. L-glutamate is the major excitatory neurotransmitter in the CNS. Glutamatergic neurotransmission is involved in most aspects of normal brain function and is perturbed in many neuropathologic conditions [24]. mGluR2 is thought to be functionally involved in cognitive disorders, drug addiction, psychosis, schizophrenia, anxiety, cerebral ischemia, and epilepsy [24–27]. Studies have shown that mGluR2 positive allosteric modulators (PAMs) could be a promising treatment for schizophrenia, and negative allosteric modulators (NAMs) have potential as antidepressant drugs [24, 28–30]. In the future, it would be valuable to clarify whether RABV G can mimic ligands or modulators (PAMs and NAMs) to affect the physiological function of mGluR2.

Metabotropic glutamate receptors (mGluRs) belong to the class of seven-transmembrane domain receptors and are classified into three groups: group I (mGluR1 and 5), group II (mGluR2 and 3), and group III (mGluR4, 6, 7, and 8) [24, 31]. Each mGluR comprises an N-terminal bi-lobed ligand-binding domain (LBD), a cysteine rich domain (CRD), and links to a G-protein-activating seven-transmembrane domain (7TMD). mGluRs are obligatory dimers; either homo- or heterodimerization of full-length mGluRs is required for G protein activation [32, 33]. mGluR homo- and heterodimerization depends primarily on an interaction at a hydrophobic interface in the upper lobe of the LBD, with modest contributions from an intersubunit disulfide bridge and a TMD interaction [33]. Subunits from distinct mGluRs can form heteromers, such as mGluR2/mGluR4 [34] or mGluR2/mGluR3 [33]. In this study, we showed that a direct interaction exists between mGluR2 and RABV G and that the mGluR2 ectodomain soluble protein effectively neutralizes cell adapted RABV *in vitro* and street RABV infections *in vivo*. Further studies are warranted to determine whether the interaction between RABV G and the mGluR2 homodimer has a different function to that of the interaction between RABV G and the mGluR2 heterodimer in RABV infection. Given the sequence and structure homology of mGluR family members, it is also important to test whether other mGluR family members function as receptors for RABV. Future studies to determine the functional mGluR2 domain that is required for RABV infection would also be informative and useful for mGluR-based RABV antiviral target discovery.

The endocytosis and recycling pathway used by some GPCRs, such as adrenergic and dopaminergic receptors, have been well studied. Typically, these receptors undergo agonist-induced endocytosis, which can be clathrin-dependent or clathrin-independent [35–37]. However, the mechanisms involved in the trafficking of mGluRs are not fully characterized. Studies to date have mainly focused on group I mGluRs, which use ß-arrestin-mediated, clathrin-dependent endocytosis to enter cells, and then undergo recycling or enter a degradation pathway [38]. However, the trafficking of group II and group III mGluRs remains largely unexplored. Indeed, only one study has reported that mGluR7 traffics via an Arf6 endosomal pathway, which is clathrin-independent [37]. RABV uses clathrin-dependent endocytosis to enter cells. Our results demonstrated that RABV and mGluR2 are internalized into cells and transported to early and late endosomes together, which may indicate that mGluR2 uses a similar endocytic pathway and endosomal traffic route in nature. However, it remains to be explored how RABV becomes internalized after binding to the receptors at the neuron membrane.

Previous studies have suggested that RABV binds and uses multiple host cellular membrane proteins to enter cells [6]. In this study, IHF confirmed the co-localization of mGluR2 and RABV antigen in brain cells of mice infected by street virus (S6 Fig). In addition, we observed that some RABV-infected brain cells were negative on mGluR2 IHF staining. This observation could mean that some brain cells expressed mGluR2 at a very low level, below the IHF detection limit, or that those cells indeed do not express mGluR2. Therefore, we cannot exclude the possibility that RABV exploits multiple cellular membrane proteins (other than mGluR2) as receptors to infect cells *in vivo*.

Many questions still remain to be address in future. Does mGluR2 and the other known receptors or as yet unidentified entry factors bind to RABV and become internalized simultaneously or sequentially? How are these cell membrane proteins used and coordinated spatially and temporally by RABV? The answers to these questions will push the boundary of our understanding of RABV pathogenesis. Our findings presented here open a new door to explore the fundamental molecular mechanism for rabies neuropathogenesis.

## Materials and methods

### Animal ethic statements

This study was carried out in accordance with the recommendations in the Guide for the Care and Use of Laboratory Animals of the Ministry of Science and Technology of China [39]. The protocols were reviewed and approved by the Committee on the Ethics of Animal Experiments of the Harbin Veterinary Research Institute of the Chinese Academy of Agricultural Sciences. Mouse challenge experiments with virulent rabies virus were conducted within the animal biosafety level 3 facilities in the Harbin Veterinary Research Institute of the Chinese Academy of Agricultural Sciences (CAAS) (approval number IACUC-2017-078).

### Cells

Human embryonic kidney (HEK293) (ATCC CRL-1573) cells and Mouse neuroblastoma cells (N2a cells) (ATCC CCL-131) were maintained in DMEM supplemented with 10% FCS, L-glutamine, and penicillin-streptomycin. Human neuroblastoma cells SK-N-SH (SK cells) (ATCC HTB-11) were maintained in MEM/EBSS supplemented with 10% FCS, L-glutamine, and penicillin-streptomycin. BSR-T7/5 cells were maintained in DMEM supplemented with 5% FCS, L-glutamine, and penicillin-streptomycin. Mouse primary neuron (mPN) cells were prepared following the established protocol; briefly, newborn mice (within 3 days of birth) were sacrificed, mouse brain was removed, and the cortex was separated at an SZM microscopic work station (Belona Technology, Hubei, China). Brain cortex was washed 3 times with PBS and cut into small pieces in 0.5 mL of PBS. Then 5 mL of trypsin was added to digest the cortex for 5 min at 37°C, after which 5 mL of DMEM supplemented with 10% FCS containing 100 μg/mL DNase was added to the cells for another 2 min. A 1-mL pipette tip was used to disperse the cells until no clots were seen. The cells were filtered through a cell strainer (BD Falcon, CA, USA) and cultured in 10 mL of Neurobasal media (ThermoFisher, Waltham, MA) supplemented with 10% B27 (ThermoFisher, Waltham, MA), 2 mM glutamine, 25 mM HEPES and 25 μg/mL ß-D-arabinofuranoside. The cells were cultured for 4 to 6 days before use.

### Viruses

RABV ERA strain was maintained in our laboratory[40]. Recombinant ERA expressing eGFP (ERA-eGFP), recombinant ERA expressing mCherry, in which the ERA *N* and *mCherry* in-frame fusion gene was inserted between the ERA *M* and *G* genes as an additional transcription unit (ERA-N-mCherry), recombinant vesicular stomatitis virus (VSV) expressing eGFP (VSV-eGFP) and VSV∆G-eGFP-ERAG, a recombinant VSV-vectored virus expressing ERA glycoprotein (G) and eGFP, in which the VSV G gene was substituted by the ERA G gene, were generated as described previously [20]. Rabies street virus GX/09[41] was passaged in mouse brain and titrated to determine the intramuscular 50% lethal dose (LD_50_) in mice before the challenge study.

### RNA interference and overexpression assays

To knockdown the expression of the mGluR2 genes in HEK293 cells, SK cells, and N2a cells, Ambion Silencer Selected siRNA (ThermoFisher, Waltham, MA, USA) was used. Briefly, siRNA (sense: 5’-CGAUUGGACG

AAUUCACUUTT-3’, antisense: 5’-AAGUGAAUUCGUCCAAUCGGT-3’, 200 nM, 5 uL per well, Ambion catalog no. s6195) targeting the mGluR2 and RABV L genes (sense: 5’-GG

AAUGCACUUUCGAUAUATT-3’, antisense: 5’-UAUAUCGAAAGUGCAUUCCTT-3’, 200 nM, 5 uL per well) or non-targeting siRNA (as a negative control, Ambion catalog no. 4390843) was mixed with 35 μL of OptiMEM medium (Invitrogen, OR, USA) containing 0.15 μL of Lipofectamine RNAiMAX transfection reagent (Invitrogen, OR, USA) on 96-well cell carrier plates (PerkinElmer, Waltham, MA, USA). After a 30-min incubation at room temperature, 60 μL of OptiMEM medium containing 1×10^4^ cells was added to the wells. The cells were incubated for 48 h to knockdown gene expression before infection with viruses. For quantitative real-time PCR (qRT-PCR), the Power SYBR Green RT-PCR kit (Applied Biosystems, Carlsbad, CA, USA) and mGluR2-specific primers (5’-gcacaggcaaggagacagc-3’ and 5’-gaggcagccaagcaccac-3’) were used to determine the mRNA copy number at 18 h post-transfection by following the manufacturer’s instructions. siRNA-silenced cells were infected with ERA-eGFP, at a multiple of infection (MOI) of 0.01. At 48 h post-infection, cells were fixed with 3% paraformaldehyde and stained with Hoechst 33342 (Invitrogen, OR, USA) in PBS for 1 h. Stained cells were imaged by using the PerkinElmer Operetta high-content system (PerkinElmer, Waltham, MA, USA). Uninfected cells served as the reference population for background fluorescence. Fifty-two fields per well were imaged at 20× magnification. Columbus software (PerkinElmer, Waltham, MA, USA) was used to automatically identify and quantify green fluorescence and cell nuclei. The infection ratio was determined according to the numbers of infected cells versus the total number of cells per well. The assay was independently repeated three times.

For the overexpression assays, HEK293 cells were seeded onto 24-well plates at 1.0×10^5^ cells per well. The cells were transfected with 0.5 μg of plasmids pCAGGS, and pCAGGS expressing mGluR2, respectively, with TransIT-293 transfection reagent (Mirus Bio, Madison, WI, USA) according to the manufacturer’s instructions. At 48 h post-transfection, the mGluR2-transfected cells were washed with DMEM three times and infected with ERA-eGFP at an MOI of 5 for 1 h at 4°C. The infected cells were incubated at 37°C, and the viral titers at different times post-infection in the supernatants were determined in BSR-T7/5 cells. The viral titer was expressed as focus forming unit per milliliter (FFU/mL).

### Cell viability assay

Cell viability was determined by using the CellTiter-Glo kit (Promega, Madison, WI, USA). Briefly, HEK293, SK, N2a, or mPN cells were seeded onto 96-well plates with opaque walls. mGluR2 monoclonal antibody A1 (sc271654; Santa Cruz Biotechnology, CA, USA) or its isotype antibody purified mouse IgG2a (0103–01, Southern Biotech, AL, USA) was added at a concentration of 40 μg/ml (expect for SK cells, for which the concentration used was 5 μg/ml). At 48 h post-addition, 100 μl of CellTiter-Glo reagent (Promega, Madison, WI, USA) was added directly into each well and incubated with the cells for 10 min on a shaker to induce cell lysis. Luminescence was measured with a GloMax 96 Microplate Luminometer (Promega, Madison, WI, USA).

### Cell plasma membrane protein isolation

Cell plasma membrane protein was isolated from 5×10^6^ cells by using the Minute plasma membrane protein isolation and cell fractionation kit (SM-005, Invent Biotechnologies, Plymouth, MN, USA) and following the manufacturer's instructions. The pellet containing plasma membrane proteins was collected to analyze for mGluR2 expression by western blotting. Na+/K+ ATPase (ab76020, Abcam, Cambridge, UK) was used as plasma membrane loading control.

### Antibody blocking

Experiments were performed on HEK293 cells, SK cells, N2a cells, and mPN cells with a mouse monoclonal antibody (mAb) to human mGluR2 A1 (sc271654; Santa Cruz Biotechnology, CA, USA) and a goat polyclonal antibody (pAb) to mGluR2 (sc47135; Santa Cruz Biotechnology, CA, USA). Mouse IgG2a (0103–01, Southern Biotech, AL, USA) and goat polyclonal IgG (ab37373, Abcam, Cambridge, UK) were used as isotype controls, respectively. Cells on 96-well carrier plates were incubated with 0.03 mL of medium containing different concentrations of antibodies and isotype antibody (40 μg/mL) at 4°C for 1 h before infection. After three washes with medium containing the corresponding antibodies, viruses at different MOIs (ERA-eGFP at 0.05, VSV∆G-eGFP-ERAG and VSV-eGFP at 0.01) were incubated with the cells at 4°C for 1 h. After another round of three washes, the cells were again incubated with medium containing the corresponding antibodies at 37°C. At 48 h after infection (16 h for VSV∆G-eGFP-ERAG and VSV-eGFP), the cells were fixed with 3% formaldehyde, and the infection ratio was determined by using the PerkinElmer Operetta high-content system. The relative infection ratio of the antibody-treated groups was calculated according to the normalized isotype control infection ratio. ERA-eGFP growth kinetics were determined by sampling the supernatant of the antibody-treated HEK293 cells or mPN cells at different times after infection and titrating the viral titers on BSR-T7/5 cells.

### Infectivity neutralization assays

The N-terminal GST-tagged soluble ectodomain of mGluR2 (mGluR2-GST, amino acids 19–567) was expressed and purified by FriendBio Technology (Wuhan, Hubei, China). HEK293 cells, SK cells, N2a cells, and mPN cells were seeded onto 96-well carrier plates. Viruses at different MOIs (ERA-eGFP at 0.05; VSV∆G-eGFP-ERAG and VSV-eGFP at 0.01) were mixed thoroughly with different concentrations of mGluR2-GST in 0.03 mL of cell culture medium at 4°C for 1 h before being used to infect the cells. The virus-protein mix was added to the cells and incubated at 37°C. At 48 h post-infection (16 h for VSV∆G-eGFP-ERAG and VSV-eGFP), the cells were fixed with 3% paraformaldehyde. Data were acquired by using the PerkinElmer Operetta high-content system and analyzed by using Columbus software. The relative infection ratio was calculated as described above.

### Co-immunoprecipitation (Co-IP) and pull-down assays

For Co-IP, plasmid pCAGGS expressing C-terminal Flag (DYKDDDDK)-tagged mGluR2 (mGluR2-Flag, GenBank accession no. NM_000839.4), CD20 (CD20-Flag, GenBank accession no. NM_152866.2), SLAM (SLAM-Flag, GenBank accession no. NM_001040288.2) and C-terminal Myc (EQKLISEEDL)-tagged ERA G (ERAG-Myc, GenBank accession no. J02293.1), CVS G[42] (CVSG-Myc,), GX/09 G (GX/09G-Myc, GenBank accession no. GQ472537.1) and WCBV G (WCBVG-Myc, GenBank accession no. EF614258.1), and VSV G (VSVG-Myc, GenBank accession no.J02428.1) were respectively co-transfected into HEK293 cells with TransIT-293 transfection reagent following the manufacturer’s instructions. At 48 h post-transfection, the cells were washed with PBS and lysed with 1% NP-40 PBS buffer for 1 h at 4°C. Cell lysates were centrifuged (12000 rpm) for 20 min at 4°C to remove cell debris. Then, 100 μL of supernatant was removed and mixed with 40 μL of protein G agarose (Roche Diagnostics GmbH, Mannheim, Germany) for 4 h at 4°C on a flip shaker. The protein G beads were then removed by centrifugation, and the supernatant was collected and mixed with anti-Flag antibody-conjugated agarose beads (Sigma-Aldrich, MO, USA) for 6 h at 4°C on a flip shaker. After conjugation, the beads were washed 5 times with pre-chilled 1% NP-40 PBS buffer. Finally, the beads were re-suspended in PBS and protein sample loading buffer, and were subjected to SDS-PAGE. Protein bands were detected by western blotting using an anti-Flag mAb (Genscript, Nanjing, China) and an anti-Myc mAb (Genscript, Nanjing, China). The Co-IP assay of ERA G and mGluR2 under acidic conditions was carried out using the same method as above, except that the pH of the lysing buffer was adjusted to 5.5.

For the ERAG and mGluR2 pull-down assay, N-terminal His-tagged ERA G protein ectodomain soluble protein (amino acids 41–450, ERAG-His) was expressed and purified from *E*. *coli* by FriendBio Technology (Wuhan, Hubei, China). mGluR2-GST or GST (10 μg) was mixed with 100 μL of Glutathione Sepharose 4B beads (GE Healthcare Bio-science, Uppsala, Sweden) for 2 h at 4°C on a flip shaker. The beads were then washed 3 times with 0.5% NP-40 PBS buffer, and re-suspended in 100 μL of PBS. Then, 5 μg of ERAG-His was added to the beads and mixed for 2 h at 4°C on a flip shaker. After conjugation, the beads were washed 5 times with 1% NP-40 PBS buffer. After the final wash, the beads were re-suspended in PBS and protein sample loading buffer. The samples were then subjected to SDS-PAGE, and assessed by western blotting using an anti-GST mAb (Genscript, Nanjing, China) and an anti-His mAb (Tiangen, Beijing, China) and the corresponding HRP-labeled secondary antibody. For the mGluR2 and other RABV G or VSV G pull-down assays, 10 μL of mGluR2-GST- or GST-loaded Glutathione Sepharose 4B beads was added to 100 μL of lysates from cells transfected with pCAGGS expressing Myc-tagged RABV G or VSV G (prepared as described above) and mixed for 6 h at 4°C on a flip shaker. After conjugation, the beads were washed, re-suspended and subjected to SDS-PAGE, and assessed by western blotting using an anti-GST mAb and an anti-Myc mAb as described above. The pull-down assay of ERA G and mGluR2 under acidic conditions was carried out using the same method as above, except that the pH of the lysing buffer was adjusted to 5.5.

### Flow cytometry

HEK293 cells were seeded onto 6-well plates at 1×10^6^ cells per well. The cells were then infected with RABV ERA at an MOI of 5 at 37°C for 1 h. Then, the cells were thoroughly washed with PBS and trypsinized with 0.25% trypsin (without EDTA), and the dispersed cells were collected in a 1.5-mL tube. The dispersed cells were washed three times with FACS wash buffer (PBS containing 2% FCS) and fixed with 3% paraformaldehyde at room temperature for 15 min. The fixed cells were washed three times and stained with mGluR2 mAb A1 as the primary antibody and FITC-conjugated rabbit anti-mouse IgG (Sigma-Aldrich, MO, USA) as the secondary antibody. Uninfected cells stained with mouse IgG2a isotype antibody served as a control. All cells were analyzed by using a FC500 flow cytometer (Beckman Coulter, Indianapolis, IN, USA). Cell surface fluorescence density was measured and analyzed by using FlowJo software (FlowJo LLC, Ashland, OR, USA).

### Multiplex immunofluorescence and Imaris 3D rendering

Multiplex immunofluorescence with Tyramide Signal Amplification (TSA) was performed in a stepwise fashion. Briefly, samples were fixed in pre-cooled 3% paraformaldehyde in PBS for 20 minutes at 4°C. Endogenous peroxidase activity was quenched by 0.3% hydrogen peroxide in PBS for 20 minutes at room temperature. After the permeabilization (0.25% Triton X-100 in PBS for 15 min) and blocking steps (ZLI-9056, Zsbio, Beijing, China), samples were incubated with primary antibody at 4°C overnight followed by HRP-conjugated secondary antibody (PV-6001/6002, Zsbio, Beijing, China) at room temperature for 30 min. TSA amplification reagent diluted 1:100 in reaction buffer (NEL811001KT, PerkinElmer, Waltham, MA, USA) was added to the sample and incubated for 30–120 sec until the best signal intensity and signal-to-noise ratio was achieved. The primary and secondary antibodies were then removed by incubation with stripping buffer at 37°C for 30 min while retaining the TSA signal. After a brief rinse, other antigens were serially detected using spectrally different TSA reagents following the above method. The primary antibodies used in this study were mouse anti-mGluR2 antibody (sc271654; Santa Cruz Biotechnology, CA, USA), rabbit anti-mCherry antibody (ab183628, Abcam, Cambridge, UK), rabbit anti-Rab5 antibody (3547, Cell Signaling Technology, MA, USA), rabbit anti-Rab7 antibody (ab137029, Abcam, Cambridge, UK), rabbit anti-Histone H3 antibody (4499, Cell Signaling Technology, MA, USA), rabbit anti-Tomm20 antibody (ab186734, Abcam, Cambridge, UK), rabbit anti-alpha tubulin antibody (ab179484, Abcam, Cambridge, UK), and rabbit anti-AIF antibody (ab32516, Abcam, Cambridge, UK). The secondary antibodies were HRP-conjugated anti-rabbit IgG (A00098, GenScript, Nanjing, China) and HRP-conjugated anti-mouse IgG (A00160, GenScript, Nanjing, China). Images were acquired using a Zeiss LSM880 laser-scanning confocal microscope (Carl Zeiss AG, Jena, Germany) with Airyscan (Plan-Apochromat, objective 63×, Numerical Aperture 1.4). Cells were scanned 24 layers along the Z axis with a pixel dwell time of 1 microsecond. The resolution of the acquired image was 2048 × 2048.

Data were processed using Bitplane Imaris software (Bitplane AG, Zurich, Switzerland) to generate 3D rendered images. Briefly, first, the red channel (RABV) and purple channel (Rab5 or Rab7) were processed using the "surface module". Then, the surface results of the purple and red channels were inputted as "cell" and "nuclei" respectively under the "cell module". Next, the green channel (mGluR2) was processed using the "surface module". After that, a new channel was established by merging the red and green channels using the "mask dataset" of the "coloc" module. Spots that represented the co-localization of RABV and mGluR2 were counted in the merged channel by using the "spot" module. Finally, the spots results were inputted into "cell", and the RABV-mGluR2 spots that co-localized with Rab5 or Rab7 (displayed as white spots in the 3D rendered image) were counted.

### Mouse studies

Randomization and blinding were used for the allocation of animals to experimental groups. Five-week-old C57BL/6 (B6) mice were obtained from Vital River Laboratories (Vital River Laboratories, Beijing, China). All procedures were carried out by trained personnel in a biosafety level 3 facility at Harvin Veterinary Research Institute (HVRI), Chinese Academy of Agricultural Sciences (CAAS). All animals were housed in cages, under controlled conditions of humidity, temperature, and light (12-h light/12-h dark cycles). Food and water were available *ad libitum*.

For the intramuscular virus challenge, 10 MLD_50_ of RABV GX/09 was mixed with different amounts of mGluR2-GST (200, 100, 50, 25, 0 μg/mL) or GST (200 μg/mL) in 0.1 mL of PBS. For the intracerebral virus challenge, 5 MLD_50_ of GX/09 was mixed with different amounts of mGluR2-GST (200, 100, 50, 25, 0 μg/mL) or GST (200 μg/mL) in 0.03 mL of PBS. The virus-protein mix was incubated on ice for 1 h before being used to challenge B6 mice by intramuscular or intracerebral inoculation. Mice intramuscularly inoculated with 0.1 mL of 200 μg/mL mGluR2-GST or intracerebrally inoculated with 0.03 mL of 200 μg/mL mGluR2-GST were used as toxicity controls. All mice were observed for 21 days for signs of sickness and death. Survival rates were generated by using GraphPad Prism software. Statistical significance was analyzed by using the software built-in Log-rank (Mantel-Cox) test.

### Immunohistochemistry and immunohistofluorescence

B6 mice were intramuscularly challenged with 10 MLD_50_ of RABV GX/09 in 0.1 mL of PBS. Mice were euthanized at the onset of neurological symptoms; whole mouse brain was removed and fixed in 10% formaldehyde/PBS (v/v) fixation buffer. The fixed brains were then dehydrated and embedded in paraffin wax and sectioned at 1.5-μm thickness. After deparaffinization and rehydration, antigen retrieval was performed by immersing the sections in a citrate acid (pH 7.4)/sodium citrate buffer solution (pH 8.0) at 121°C for 30 min. Endogenous peroxidase activity was quenched by a 3% H_2_O_2_ methanol solution for 30 min at room temperature.

After thorough washing, the sections were blocked with 5% skim milk (Sigma-Aldrich, MO, USA) in PBS for 30 min at room temperature.

For immunohistochemistry, after being incubated with 1:100 diluted rabbit anti-mGluR2 antibody (ab150387, Abcam, Cambridge, UK) or 1:10 diluted monoclonal anti-RABV P protein antibody (prepared in our laboratory) overnight at 4°C, the sections were incubated with horseradish peroxidase-conjugated goat anti-rabbit secondary antibody (for mGluR2) or goat anti-mouse secondary antibody (for RABV) for 30 min at room temperature, rinsed three times with PBS for 5 min each, rapidly visualized using diaminobenzidine (DAB) (Sigma-Aldrich), and counterstained with hematoxylin. The sections were then dehydrated using ascending concentrations (70%, 95%, and 100%) of ethanol, cleared in xylene, and mounted with Entellan mounting medium (Sigma-Aldrich). Staining was observed using a light microscope.

For immunohistofluorescence, the sections were incubated with 1:100 diluted rabbit anti-mGluR2 antibody (ab150387, Abcam, Cambridge, UK) overnight at 4°C, then washed and incubated with 1:100 diluted fluorescein goat anti-rabbit IgG (FI-1000, Vector Labs, Burlingame, CA, USA) in the dark at room temperature for 30 min. Subsequently, the sections were incubated with 1:10 diluted monoclonal anti-RABV P protein antibody (prepared in our laboratory) overnight at 4°C, then washed and incubated with 1:100 diluted DyLight 680-labeled anti-mouse IgG (5230–0344, SeraCare, Milford, MA, USA) in the dark at room temperature for 30 min. After a final wash, the sections were stained with Fluoroshield with DAPI (F6057, Sigma-Aldrich, MO, USA) before being mounted. Slides were imaged using a Carl Zeiss LSM700 microscope (Carl Zeiss, Heidenheim, Germany).

## Supporting information

S1 Fig HEK293, SK, and N2a cells express mGluR2. Cell plasma membrane extracts of HEK293, SK, and N2a cells were subjected to western blotting to detect mGluR2 expression (A). Surface expression of mGluR2 om HEK293 (B), SK (C), and N2a (D) cells was confirmed by flow cytometry.
(TIF)

S2 Fig RABV G does not interact with SLAM or CD20. SLAM-Flag or CD20-Flag was co-immunoprecipitated with ERAG-Myc in plasmid-transfected HEK293 cell lysates. No interaction was detected between ERA G and SLAM or CD20.
(TIF)

S3 Fig Cell viability is unaffected by mGluR2 monoclonal antibody treatment. HEK293 (A), N2a (C), and mPN (D) cells were treated with 40 μg/ml mGluR2 monoclonal antibody or its isotype antibody purified mouse IgG2a. SK cells (B) were treated with 5 μg/ml mGluR2 monoclonal antibody A1 or purified mouse IgG2a. Cell viability was determined by using the CellTiter-Glo luminescent cell viability assay kit (Promega, Madison, WI, USA). No statistically significant differences in viability were observed between the mGluR2 monoclonal antibody- and IgG2a-treated cells.
(TIF)

S4 Fig Antibodies against mGluR2 block RABV infection of cells. The monoclonal antibody (mAb) or polyclonal antibody (pAb) against mGluR2 blocked ERA-eGFP infection of HEK293 cells (A, B) and mPN cells (C).
(TIF)

S5 Fig The mGluR2 ectodomain soluble protein (mGluR2-GST) neutralized the infectivity of RABV. mGluR2-GST neutralized ERA-eGFP infection of HEK293 cells (A) and mPN cells (B).
(TIF)

S6 Fig Immunohistochemistry and immunohistofluorescence of brain sections from mice challenged with street virus GX/09. B6 mice were intramuscularly challenged with 10 MLD_50_ of GX/09. Whole brain sections were immunohistochemically stained for mGluR2 (A) and RABV antigen (B), or fluorescently stained for mGluR2 (green) and RABV (red) (C, D, and E). Five fields from (E) were selected for detailed observation of mGluR2 and RABV antigen in cells from the brainstem (I), cerebellum (II), pons (III), cerebral cortex (IV), and olfactory bulb (V); these fields were observed under a Carl Zeiss LSM700 microscope.
(TIF)
