# Supplementary figures and images for: Metabotropic glutamate receptor subtype 2 is a cellular receptor for rabies virus
Source: PLoS Pathog. 2018 Jul 20;14(7):e1007189. doi: 10.1371/journal.ppat.1007189 (PMC6070288; doi:10.1371/journal.ppat.1007189)

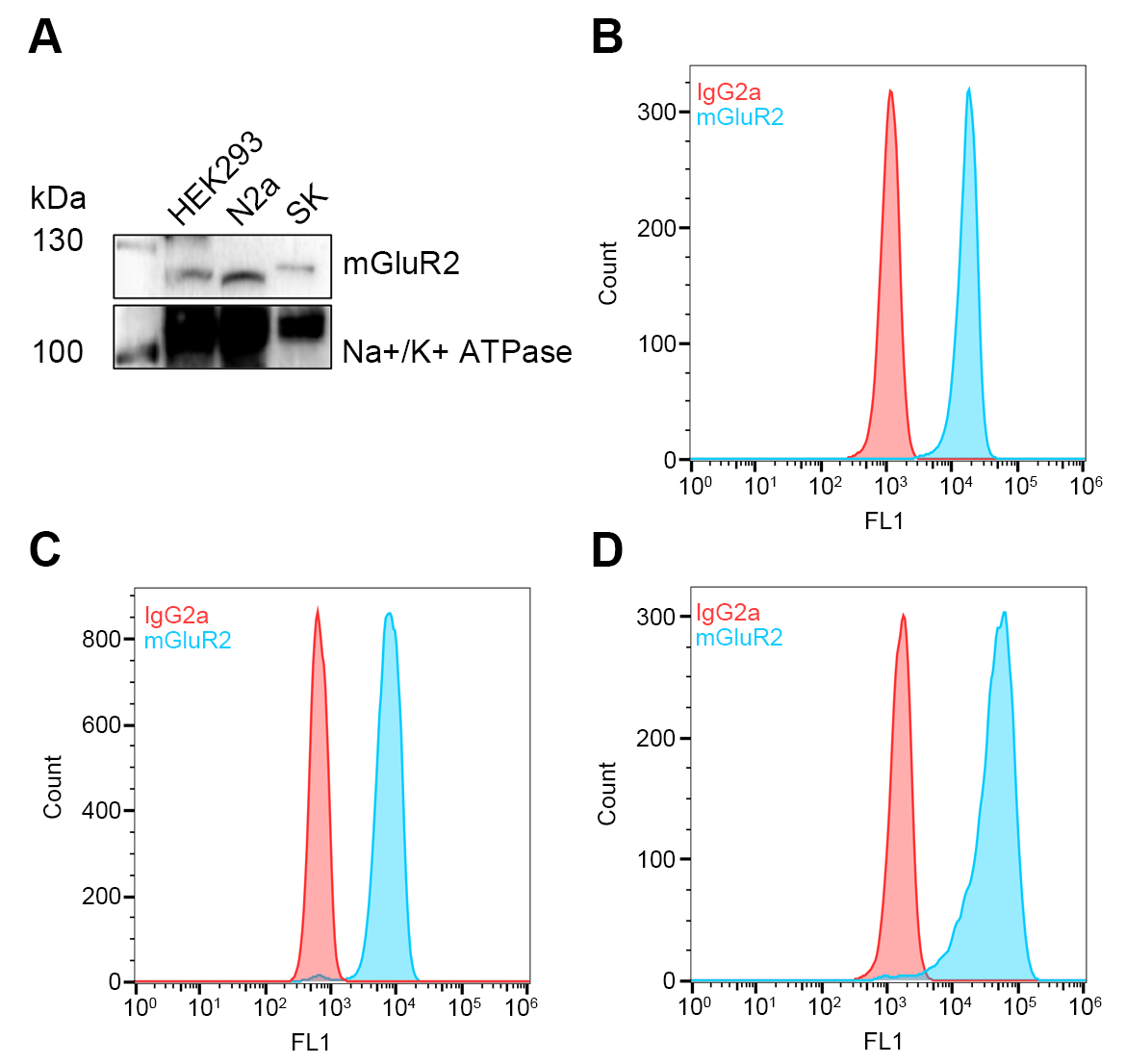

Supplement: S1 Fig — Cell plasma membrane extracts of HEK293, SK, and N2a cells were subjected to western blotting to detect mGluR2 expression (A). Surface expression of mGluR2 om HEK293 (B), SK (C), and N2a (D) cells was confirmed by flow cytometry. (TIF) [file ppat.1007189.s001.tif]

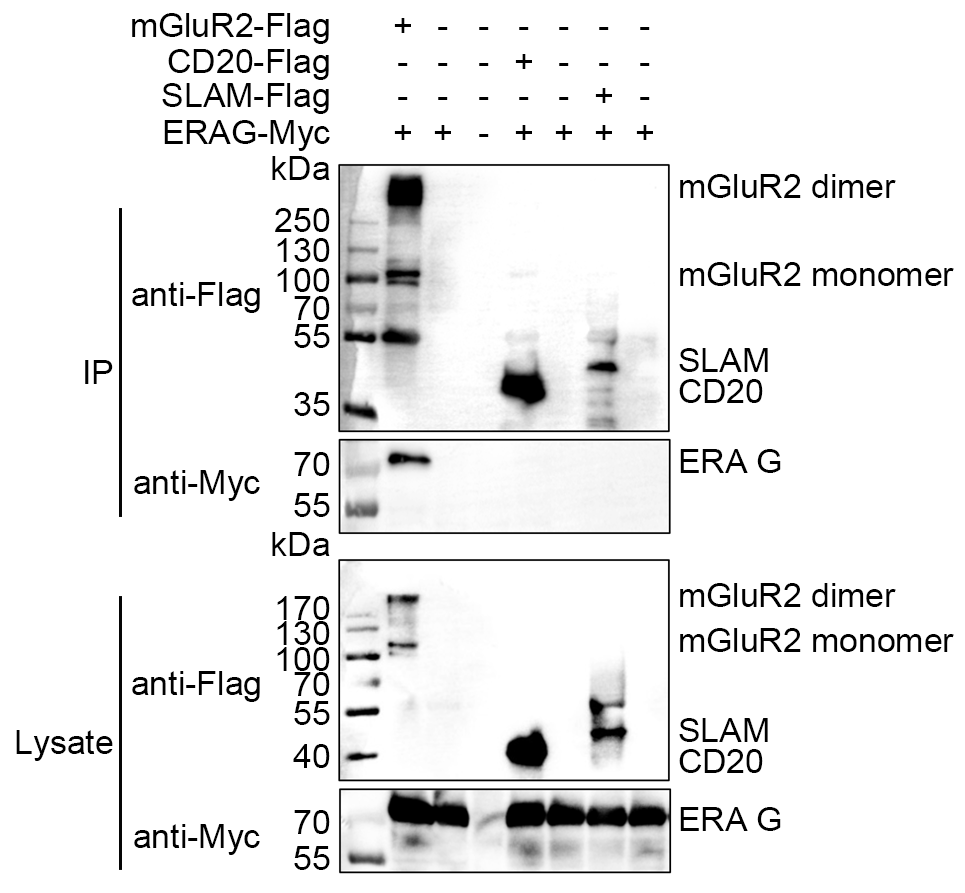

Supplement: S2 Fig — SLAM-Flag or CD20-Flag was co-immunoprecipitated with ERAG-Myc in plasmid-transfected HEK293 cell lysates. No interaction was detected between ERA G and SLAM or CD20. (TIF) [file ppat.1007189.s002.tif]

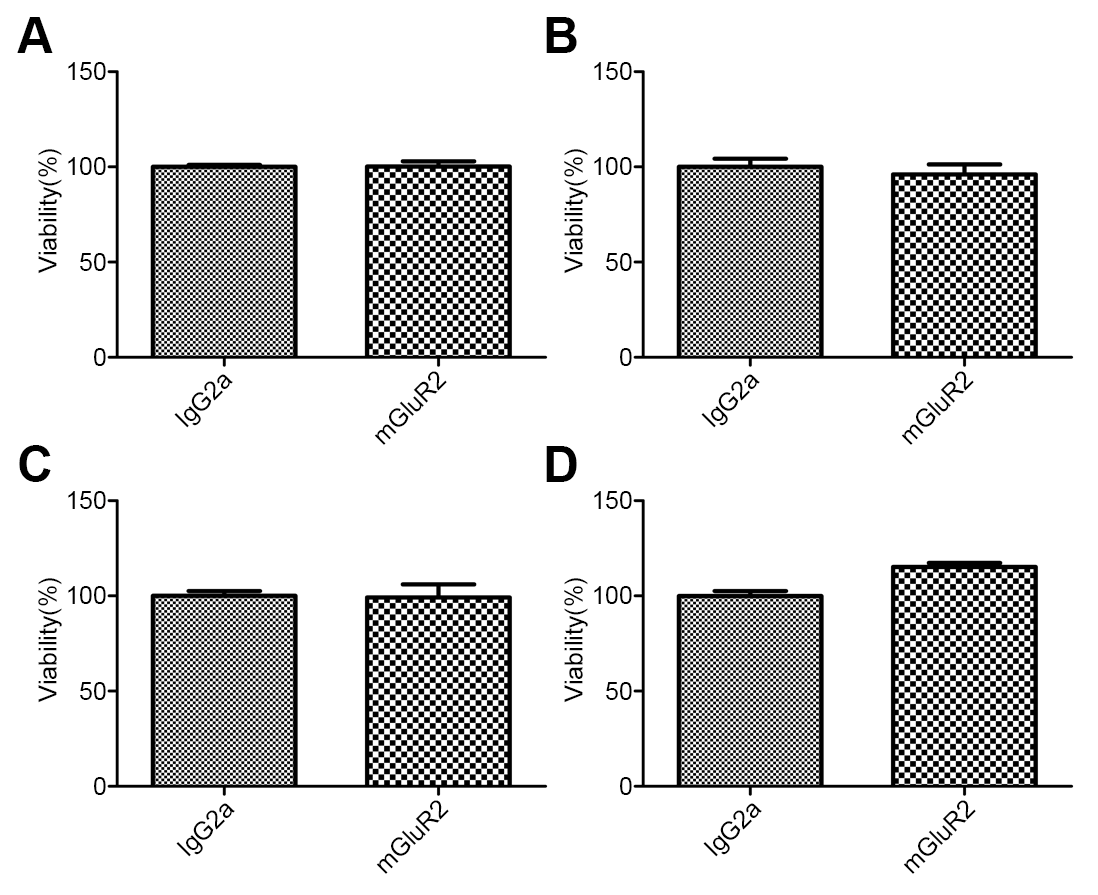

Supplement: S3 Fig — HEK293 (A), N2a (C), and mPN (D) cells were treated with 40 μg/ml mGluR2 monoclonal antibody or its isotype antibody purified mouse IgG2a. SK cells (B) were treated with 5 μg/ml mGluR2 monoclonal antibody A1 or purified mouse IgG2a. Cell viability was determined by using the CellTiter-Glo luminescent cell viability assay kit (Promega, Madison, WI, USA). No statistically significant differences in viability were observed between the mGluR2 monoclonal antibody- and IgG2a-treated cells. (TIF) [file ppat.1007189.s003.tif]

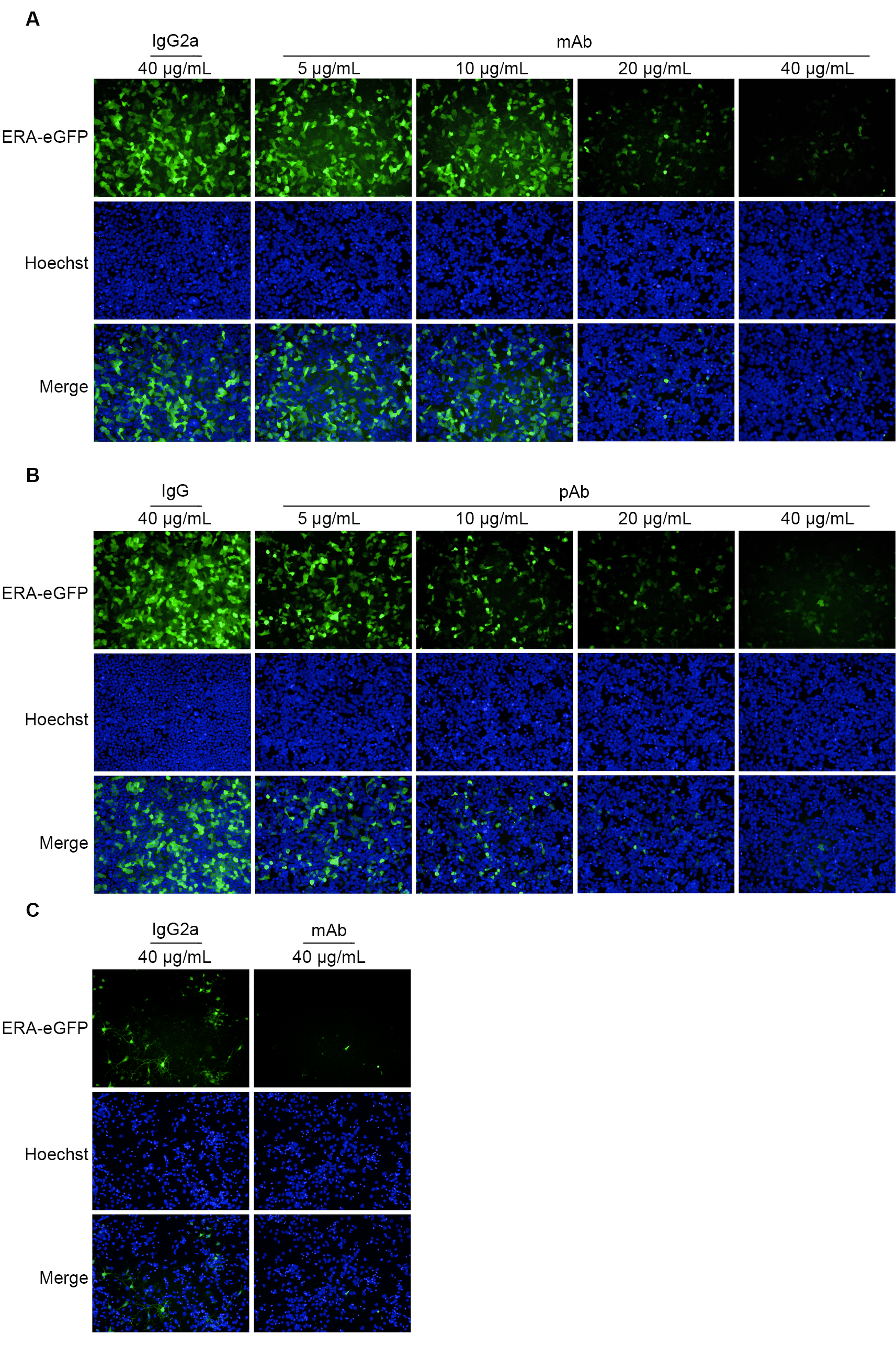

Supplement: S4 Fig — The monoclonal antibody (mAb) or polyclonal antibody (pAb) against mGluR2 blocked ERA-eGFP infection of HEK293 cells (A, B) and mPN cells (C). (TIF) [file ppat.1007189.s004.tif]

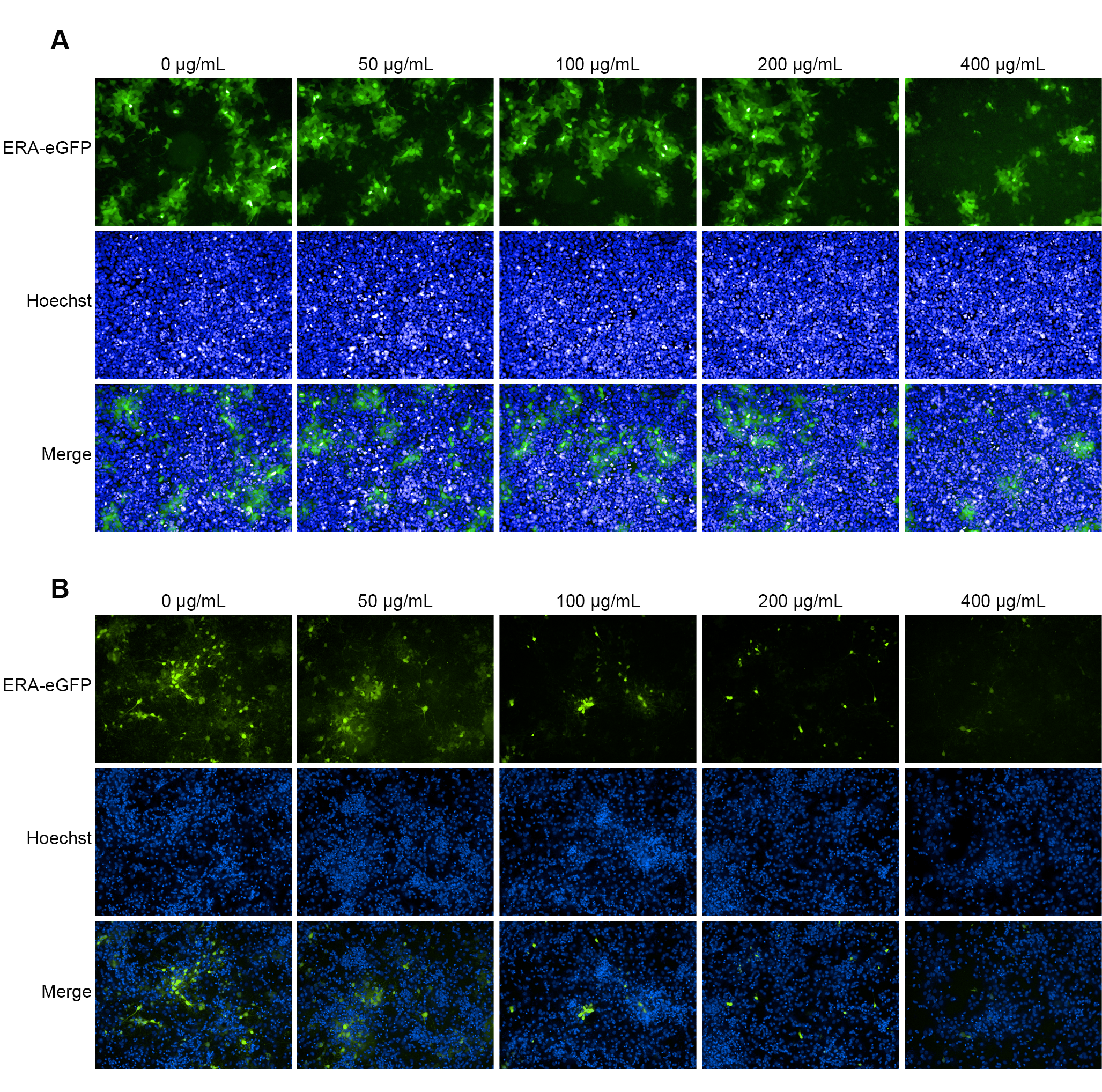

Supplement: S5 Fig — mGluR2-GST neutralized ERA-eGFP infection of HEK293 cells (A) and mPN cells (B). (TIF) [file ppat.1007189.s005.tif]

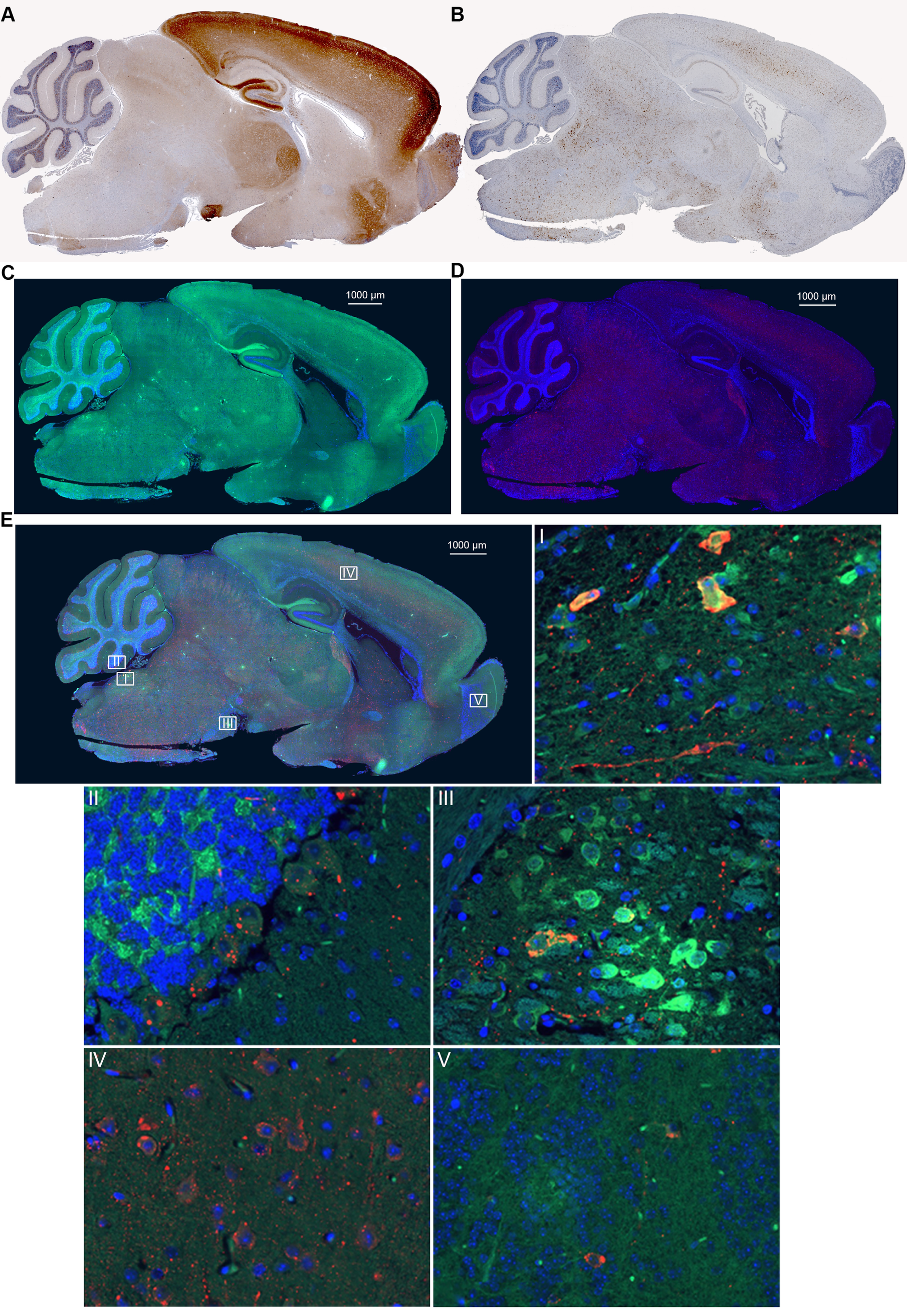

Supplement: S6 Fig — B6 mice were intramuscularly challenged with 10 MLD50 of GX/09. Whole brain sections were immunohistochemically stained for mGluR2 (A) and RABV antigen (B), or fluorescently stained for mGluR2 (green) and RABV (red) (C, D, and E). Five fields from (E) were selected for detailed observation of mGluR2 and RABV antigen in cells from the brainstem (I), cerebellum (II), pons (III), cerebral cortex (IV), and olfactory bulb (V); these fields were observed under a Carl Zeiss LSM700 microscope. (TIF) [file ppat.1007189.s006.tif]
